# The efficacy of Yiqi Huoxue therapy for chronic heart failure: A meta-analysis in accordance with PRISMA guideline

**DOI:** 10.1097/MD.0000000000030082

**Published:** 2022-08-26

**Authors:** Miao Zhang, Ming-Yue Sun, Hui-Jun Yin, Zheng-Zhi Wu, Yu Jin, Ma Min, Feng-Qin Xu

**Affiliations:** a Shenzhen Second People’s Hospital, First Affiliated Hospital of Shenzhen University, Shenzhen, China; b Jinan University, Guangzhou, China; c Department of Cardiovascular Disease, Beijing Xiyuan Hospital, China Academy of Chinese Medical Sciences, Beijing, China.

**Keywords:** chronic heart failure, meta-analysis, traditional Chinese medicine, Yiqi Huoxue

## Abstract

Chronic heart failure (CHF) is the final destination of most cardiovascular diseases and the most important cause of death. The main clinical manifestations were pulmonary congestion and decreased cardiac output. The purpose of this systematic review is to evaluate the effectiveness of Yiqi Huoxue therapy on CHF.

**Methods:**

Seven electronic databases were searched to identify randomized controlled trials of Yiqi Huoxue (YQHX) method for CHF until April 30, 2020. The quality assessment of the included trials was performed by employing the Cochrane Risk of Bias tool and Jadad scale.

**Results:**

Nineteen randomized controlled trials were included in our review. Most of the included trials were considered as low quality. The aggregated results suggested that experimental group with YQHX therapy got better effect in increasing overall response rate (risk ratio, RR = 1.21, 95% confidence interval, CI 1.15–1.27), traditional Chinese medicine (TCM) syndrome response rate (RR = 1.26, 95% CI 1.17–1.36), 6-minute walk test (RR = 2.14, 95% CI 1.05–3.22), left ventricular ejection fraction (RR = 0.97, 95% CI 0.60–1.34), and stroke volume (standardized mean difference, SMD = 0.94, 95% CI 0.23–1.56), and in lowering down the TCM syndrome scores (SMD = –0.78, 95% CI –0.91 to –0.64), Minnesota Living with Heart Failure questionnaire (SMD = –1.01, 95% CI –1.56 to –0.45), 6-month readmission rate (RR = 0.50, 95% CI 0.28–0.89), B-type natriuretic peptide (SMD = –0.89, 95% CI –1.52 to –0.25), NT-proBNP (SMD = –2.07, 95% CI –3.34 to –0.08), and C-reactive protein (SMD = –2.04, 95% CI –4.12 to –0.67) as compared to using conventional Western medicine alone. There were no significant differences found in left ventricular end diastolic diameter and E/E′ between experimental groups and control groups. Moreover, the included sample capacity is small and the trails are all in Chinese. Quality of the evidence for outcomes were “low” and “very low” according to the GRADE assessment.

**Conclusion:**

YQHX is a valid complementary and alternative therapy in the management of CHF, especially in improving overall response rate, TCM syndrome response rate, 6-minute walk test, left ventricular ejection fraction, and stroke volume and in decreasing TCM syndrome scores, Minnesota Living with Heart Failure questionnaire, 6-month readmission rate, B-type natriuretic peptide, NT-proBNP, and C-reactive protein levels. Hence, YQHX is a relatively effective and safe therapy for CHF patients, which can be popularized and applied in the clinic. More long-term follow-up studies are still needed to substantiate and confirm the current findings.

## 1. Introduction

Chronic heart failure (CHF) is a complex clinical syndrome characterized by insufficient blood perfusion of tissues and organs, abnormal distribution of peripheral blood flow, and activation of neuroendocrine, which is caused by the impairment of ejection function or ventricular filling due to the abnormality of heart function or structure.^[1,2]^ The incidence rate of CHF increases year by year. The disease seriously affects the daily life of patients, reduces the quality of life of patients, and even threatens the safety of patients.^[3]^ CHF is characterized by chest distress, wheezing, and paroxysmal nocturnal dyspnea. The specific clinical manifestations are palpitation, chest distress, shortness of breath, restlessness and insomnia, dry mouth, and dry tongue, often accompanied by poor urination, lower extremity edema, and cyanosis of lips with petechiae, etc. The condition is often lingering and difficult to recover, and prone to recurrent attacks. CHF is more common in the elderly. The main modern medical treatment includes general treatment (removing inducements, adjusting life style and oxygen inhalation, etc) and drug treatment. The commonly used drug treatment mainly includes cardiotonics, diuretics, blood pressure and rhythm control, vasodilators, etc.^[4]^ Modern medicine believes that these drugs can effectively improve myocardial contraction and reduce heart load; furthermore, it can improve hemodynamics to achieve ideal therapeutic effect. However, due to the vulnerable pathological and physiological characteristics of the elderly, adverse reactions to modern medical treatment, such as severe water and electrolyte disorders, persistent dry cough, drug poisoning, etc., may lead to serious consequences.^[5]^ Whereas, our findings show that the combination of traditional Chinese and Western medicine demonstrates its unique advantages and characteristics in treating CHF and avoiding the occurrence of some common adverse reactions.

Based on the basic theories of traditional Chinese medicine (TCM), CHF is equivalent to the term of “Xiong Bi” or “Chuan Zheng” or “Shui Zhong” or “Tan Yin”. The etiology and pathogenesis of CHF are related to Qi deficiency and blood stasis. In TCM, Qi is the vital life force in the body, supposedly able to be regulated by food and medicine. Qi is the concept of life-breath or vital energy was formulated as an indication of the awareness of man, originally directed externally toward nature or society but later turned inward to the self or life within. Qi deficiency in the body may cause poor blood circulation condition and cardiac vascular occlusion, resulting in abnormal heart function.^[6]^ Therefore, invigorating qi and activating circulation to remove blood stasis (Chinese name in pinyin is “Yiqi Huoxue”) is an important therapy for CHF.^[7]^ Yiqi Huoxue therapy (YQHX) is widely used to treat patients with CHF. A large number of animal and clinical trials have confirmed that YQHX can improve the heart function and clinical symptoms of patients with CHF by inhibiting or delaying ventricular remodeling, improving diuretic resistance, improving insulin sensitivity, regulating myocardial energy metabolism, protecting mitochondria and other mechanisms of action, so as to achieve the therapeutic effect, improve the quality of life of patients, with multichannel and multitarget effects and little side effects.^[8–11]^ Therefore, the treatment of CHF with YQHX has high clinical application value and good prospect, which is worth promoting. However, the scientific evidence of the effect of YQHX on CHF is so far unknown or the current available information is not systematic. Therefore, we conducted a meta-analysis of clinical randomized controlled trials to evaluate the efficacy and safety of YQHX on patients with CHF.

## 2. Methods

### 2.1. Search strategy

Randomized controlled trials (RCTs) assessing the administration of YQHX Oral Chinese Herbal Medicine in the treatment for CHF were located by searching the databases CNKI, WANFANG, VIP, Sinomed, PubMed, EMBase, and the Cochrane Controlled Trials Register and assisted by manual retrieval. The last search was run on October 31, 2021, and case reports and small case series were excluded. No limit was placed on the language.

PubMed searching strategy includes the following:

#1: Search “Medicine, Chinese Traditional”[Mesh];
#2: Search “Drugs, Chinese Herbal medicine”[Mesh];
#3: Search “Yiqi Huoxue”[Mesh];
#4: Search “Supplementing qi activating blood circulation”[Mesh];
#5: Search “chronic heart failure”[Mesh];
#6: Search “CHF”;
#7: Search (#1 OR #2 OR #3 OR #4) AND (#5 OR #6)

Above strategies were adopted for each specific database, and Chinese characters for relevant key words were used when searching Chinese databases.

### 2.2. Study selection

Studies were selected according to the Cochrane Handbook for Systematic Reviews of Interventions.^[12]^

#### 2.2.1. Inclusion criteria.

Studies meeting the following criteria were included:

1. the studies were performed as RCTs or quasi-randomized controlled trials^[13]^;
2. patients were diagnosed with CHF;
3. Jadad score^[14]^ ≥3;
4. Western medicine was permitted to be taken according to individual symptoms;
5. YQHX formula (have classic composition *Astragalus* or *Salvia miltiorrhiza* or *Codonopsis pilosula* or *Ginseng* with clear dose) with conventional Western medicine (CWM) was used for the experimental group and CWM alone for the control group.

CWM including interventions such as oxygen uptake, rest-cure, and low-salt diet, with medicines including angiotensin-converting enzyme inhibitors, angiotensin-receptor blockers, beta-receptor blockers, diuretics, aldosterone receptor blockers, digitalis preparation, drugs belonging to ester nitrate, and others recommended in the Chinese suggestions for diagnosis and treatment of CHF. The outcomes included 6 echo-related index: overall response rate (ORR), TCM syndrome response rate (TCMSRR) by referring to the evaluation criteria of Guidelines for clinical research on Chinese new herbal medicines^[15]^ (Table 1), TCM syndrome scores (TCMSS), Minnesota Living with Heart Failure questionnaire (MLHFQ), 6-minute walk test (6MWT), and 6-month readmission rate (6MRR); 3 serum biomarkers: levels of natriuretic peptides (BNP or NT-proBNP) and C-reactive protein (CRP); 4 cardiac function index: left ventricular ejection fraction (LVEF), left ventricular end diastolic diameter (LVEDD), E/E′, and stroke volume (SV). We classified “markedly effective and effective” as an effective result and “invalid and pejorative” as an ineffective result. The ORR is the ratio of effective cases to total cases.

**Table 1:** Evaluation criteria on the efficacy of clinical symptoms and TCM syndromes recommended by GCRNDTCM.

Classification	Detailed description
Markedly effective	Clinical symptoms and signs completely disappeared, or the score ratio of clinical symptoms/TCM syndromes reduction to ≥70%
Effective	Clinical symptoms and signs were significantly reduced, with clinical symptoms/TCM syndrome score ratio reduction to 30%, but <70%
Invalid	Clinical symptoms and signs were partially reduced, with clinical symptoms/TCM syndrome score ratio reduction <30%
Pejorative	The score ratio of clinical symptoms or TCM syndromes got worse

GCRNDTCM = Guidelines of Clinical Research of New Drugs of Traditional Chinese Medicine, TCM = traditional Chinese medicine.

#### 2.2.2. Exclusion criteria.

Trials that met the following criteria were excluded:

1. Yiqi Huoxue formula were used as the only treatment
2. the target population was incongruent with diagnostic criteria of CHF;
3. the main intervention was mixed with too many measures;
4. the study was allocated without appropriate comparator or without randomization;
5. the studies with data unavailable or duplicate publication.

### 2.3. Data abstraction

Two authors (M.Z. and M.S.) independently screened the titles and abstracts of the achieved citations from primary searching. Full text of the articles of potential interest were downloaded for further evaluation, and those meeting inclusion criteria were included in the final review. The following contents were extracted from the included trials independently by 2 authors (M.Z. and M.S.): publication data (authors, publication year, study design, randomization, Jadad score, sample size, gender, and age); treatment protocol (YQHX formula and ingredients, Western medicine name, and dose); duration of treatment; main outcomes; adverse events; and duration of follow-up. If there were discrepancies in the process of selection, whether to include or exclude a study was resolved by a third author (H.Y.)’s opinion. Missing data were achieved through contacting authors of the original studies by telephone, email, or fax.

### 2.4. Quality assessment

The methodological quality of trials was assessed independently by 2 authors (M.Z. and M.S.) using criteria from the Cochrane Handbook for Systematic Review of Interventions.^[12]^ The items included random sequence generation, allocation concealment, blinding, incomplete outcome data, selective outcome reporting, and other bias (defined as baseline data comparability). We judged each item from 3 levels (“yes” for a low risk of bias, “no” for a high risk of bias, and “unclear” otherwise). Then the methodological quality of the trials was ranked into 3 levels: low risk of bias (all items with low risk of bias), high risk of bias (at least 1 item with high risk of bias), or unclear risk of bias (at least 1 item with an unclear domain). The discrepancies were resolved through consensus. Disagreements between the 2 authors were resolved by discussion and if needed, arbitrated by a third author (H.Y.).

### 2.5. Statistical methods

Meta-analyses of RCTs were performed using RevMan 5.3 software from the Cochrane Collaboration for data analyses. Data were summarized by using risk ratio (RR) with 95% confidence intervals (CI) for discontinuous outcomes, or standard mean difference (SMD) with 95% CI for continuous outcomes. We assessed data by both fixed effect model and random effect model, but reported random effect analysis only if the heterogeneity was statistically significant.^[16]^ Statistical heterogeneity was tested by examining I^2^, meaning that an I^2^ >50% indicated the possibility of statistical heterogeneity,^[17]^ and the value of *P* < .05 was regarded as statistically significant. If heterogeneity was low (I^2^ < 50% or *P* > .05), the fixed effects model was used. If heterogeneity was high (I^2^ > 50% or *P* < .05), the random effect model was used and subgroup analyses were conducted to determine the evidence for the different control if data were sufficient. Publication bias was assessed by funnel plot analysis if the group included more than 10 trials.^[18]^

## 3. Results

### 3.1. Study selection

**Figure 1.**
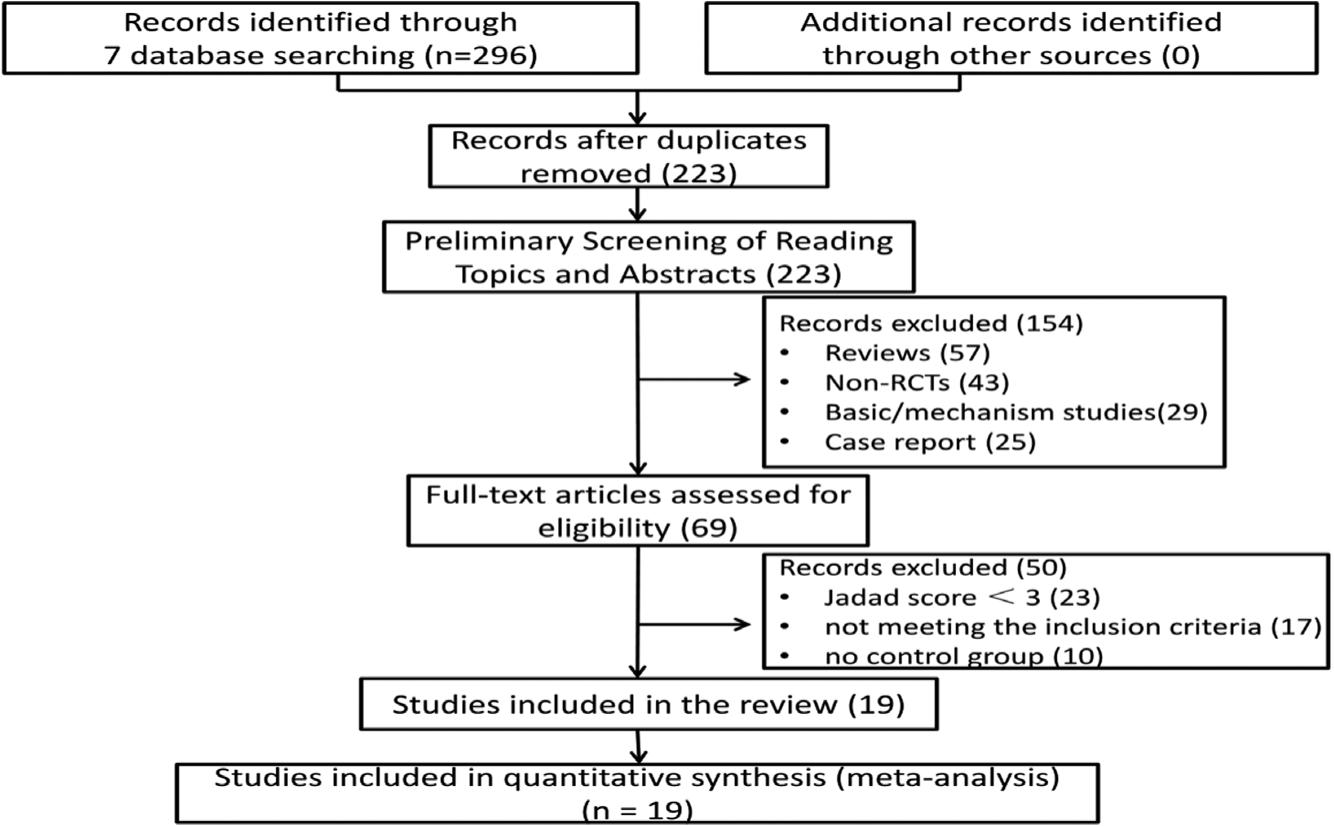
Flow diagram of study selection and identification.

The search of 7 English and Chinese databases identified 296 records for further evaluation (Fig. 1). After removing duplicates, 223 potentially relevant abstracts were initially screened, and 154 were excluded for failing to meet the inclusion criteria. We retrieved and reviewed 69 full-text articles. Fifty studies were excluded due to nonrandomized, duplicate publications, suspicion of counterfeit, and failure to get available data. Nineteen RCTs of them were eligible.^[19–37]^ No dissertations and trial registrations were obtained. All studies involved patient consent, and informed consent was given. Details of the study flow are shown in Figure 1.

### 3.2. Study characteristics

All of the 19 included trials were conducted in China and published in Chinese language. All studies were performed in China, and the studies involved a total of 2087 patients (control group 1034 patients, experimental group 1053 patients). In addition, all studies exhibited comparable baseline patient characteristics, including age and gender (male patients 1141, female patients 946). In addition, there were no significant differences among them. The characteristics of selected studies are shown in Table 2.

**Table 2:** Characteristics of included studies.

ID (author/year)	Case (T/C)	Age (T/C)	Gender (M/F)	Control group	Intervention group	Treatment duration	Outcome	Jadad score
Liu F/2017	70/65	66.1/65.8	70/65	CWM	YQHX + CWM	28D	ORR, NT-proBNP, LVEF, 6MWT, TCMSS	3
Ye Y/2014	60/58	NA	63/55	CWM	YQHX + CWM	28D	BNP, LVEF, CRP	3
Tang S/2018	60/60	67.32/68.32	69/51	CWM	YQHX + CWM	3M	ORR, TCMSRR, NT-proBNP, LVEF, LVEDD, CRP	3
Zhou SP/2018	36/36	54/52.39	37/35	CWM	YQHX + CWM	14D	ORR, TCMSRR, 6MWT	3
He P/2018	40/40	60.5/60.1	46/34	CWM	YQHX + CWM	3M	TCMSRR, LVEF, 6MWT, LVEDD, SV	3
Yu GZ/2018	60/60	64/63	65/55	CWM	YQHX + CWM	28D	ORR, 6MWT, TCMSRR, 6MRR	3
Wang K/2018	50/50	65.32/64.27	49/51	CWM	YQHX + CWM	28D	ORR, 6MWT, TCMSRR	3
Chen WT/2017	60/60	68.37/67.52	64/56	CWM	YQHX + CWM	56D	ORR, BNP, 6MWT, TCMSRR, LVEDD, E/E′, MLHFQ	4
Zhang Y/2014	40/40	69.36/79.73	46/34	CWM	YQHX + CWM	28D	ORR, 6MWT, TCMSRR, 6MRR	3
Wang D/2015	68/68	62.34/61.83	91/45	CWM	YQHX + CWM	56D	BNP, LVEF, TCMSRR	3
Liu H/2017	132/122	73.12/72.42	136/118	CWM	YQHX + CWM	28D	ORR, LVEF, LVEDD, MLHFQ	3
Yang J/2017	43/43	65.23/64.57	44/42	CWM	YQHX + CWM	28D	NT-proBNP, LVEF, E/E′	3
Qian SQ/2018	43/43	73.56/72.89	47/39	CWM	YQHX + CWM	30D	ORR, BNP, LVEF, 6MWT, LVEDD	3
Feng XX/2013	30/30	62.4 ± 6.7	34/26	CWM	YQHX + CWM	42D	NT-proBNP, LVEF, LVEDD	3
Li HL/2019	36/36	56.67/56.43	41/31	CWM	YQHX + CWM	14D	ORR, NT-proBNP, LVEF	3
Li XX/2019	59/59	67.3/67.6	61/57	CWM	YQHX + CWM	14D	ORR, BNP, NT-proBNP, LVEF	3
He PG/2019	48/47	57.73/55.49	58/37	CWM	YQHX + CWM	56D	ORR, TCMSRR, BNP, LVEF, 6MWT, LVEDD, SV, MLHFQ, CRP	4
Wang XL/2019	80/80	52.85/51.59	83/78	CWM	YQHX + CWM	3M	BNP, TCMSRR, 6MWT, MLHFQ	3
Zhang Y/2019	37/37	60/60	37/37	CWM	YQHX + CWM	30D	ORR, BNP, LVEF, LVEDD	4

6MRR = 6-month readmission rate, 6MWT = 6-minute walk test, BNP = B-type natriuretic peptide, CRP = C-reactive protein, CWM = conventional Western medicine, LVEDD = left ventricular end diastolic diameter, LVEF = left ventricular ejection fraction, MLHFQ = Minnesota Living with Heart Failure questionnaire, NA = not applicable, ORR = overall response rate, SV = stroke volume, TCMSRR = TCM syndrome response rate, TCMSS = TCM syndrome scores, YQHX = Yiqi Huoxue.

### 3.3. Study quality

**Figure 2.**
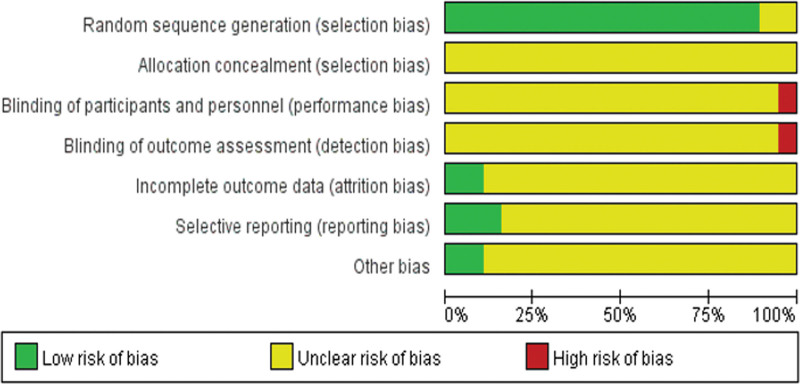
Risk of bias: reviewing authors’ judgments about each risk of bias item for each included study.

**Figure 3.**
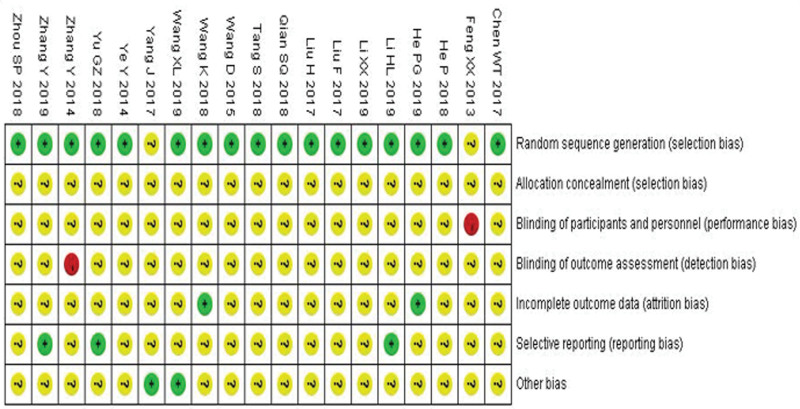
Summary of the risk of bias assessment for included trials.

Among trials, 19 studies^[19–37]^ stated the method of the sequence generation with random number table and drawing, while none of the 19 studies reported details for sample size calculations and none was double-blind, placebo controlled study. Additionally, none mentioned allocation concealment or blinding methods. There are 15 included trials^[19,21,23–29,31,33–37]^ which were assessed as low risk of bias in incomplete outcome data, since outcome data was complete. Fourteen of the included trials^[20,22–24,26,27,29–36]^ were assessed as low risk of reporting bias, and the other 5 trials^[19,21,25,28,37]^ was evaluated as unclear risk of reporting bias due to the selective reporting of predefined outcomes. Among all RCTs, the characteristics of participants in each study arm were similar at baseline (age, race, sex, and disease course), then we evaluated all of the trials at an unclear risk of other bias. The details of the risk of bias of each trial are presented in Figures 2 and 3.

### 3.4. Effects of the interventions

There was no report of mortality as the primary outcome measures (e.g., AMI, severity arrhythmia, revascularization). We analyzed the outcomes: 6 echo-related index: ORR (13 trials), TCMSRR (4 trials), TCMSS (8 trials), MLHFQ (6 trials), 6MRR (2 trials), and 6MWT (8 trials); 3 serum biomarkers: BNP (9 trials), NT-proBNP (5 trials), and CRP (3 trials); 4 cardiac function index: LVEF (14 trials), LVEDD (8 trials), SV (2 trials), and E/E′ (2 trials).

#### 3.4.1. Echo-related index.

##### 3.4.1.1. Overall response rate (ORR).

**Figure 4.**
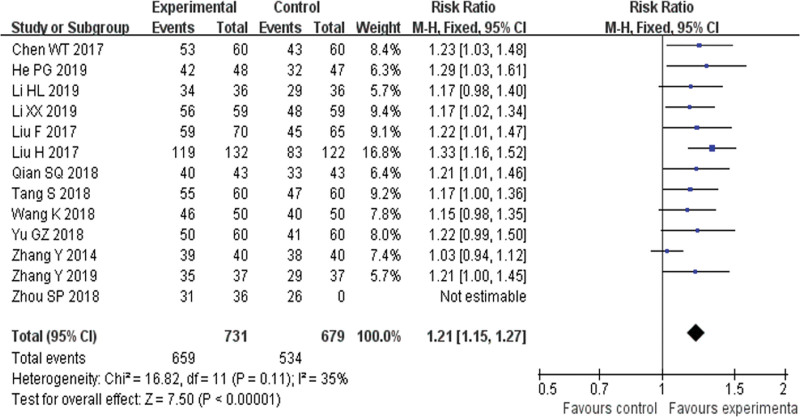
Forest plot of overall response rate.

Thirteen RCTs^[19,21–23,25–27,29–31,35–37]^ reported ORR and found an obvious difference (*P* < .00001) between YQHX plus routine Western medicine treatment and routine Western medicine treatment alone on ORR (RR = 1.21, 95% CI 1.15–1.27, 1410 participants), which meant that YQHX plus routine Western medicine treatment was significantly better than routine Western medicine treatment in acquiring better curative effectiveness. No heterogeneity was found among the 13 trials (*I*^2^ = 35%, *P* = .11) (Fig. 4).

##### 3.4.1.2. TCM syndrome response rate.

**Figure 5.**
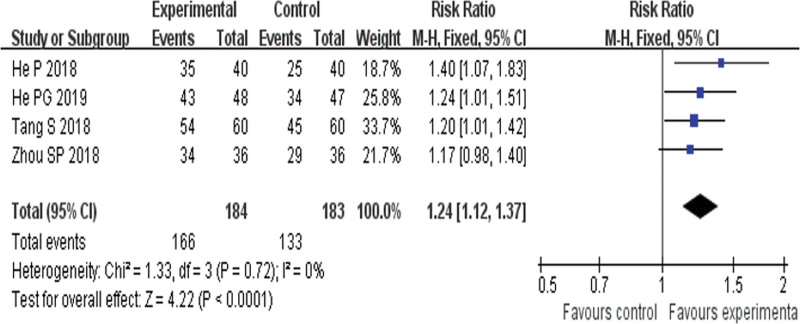
Forest plot of TCM syndrome response rate. TCM = traditional Chinese medicine.

Four RCTs^[21,22,24,27]^ reported TCMSRR and found an obvious difference (*P* < .00001) between YQHX plus routine Western medicine treatment and routine Western medicine treatment alone on TCMSRR (RR = 1.24, 95% CI 1.12–1.37, 367 participants). The result indicated that YQHX combined with conventional Western drugs group was significantly better than conventional Western drugs group in the TCMRR and there was significant homogeneity among the 4 trials (*I*^2^ = 0%, *P* = .72) (Fig. 5).

##### 3.4.1.3. TCM syndrome scores.

**Figure 6.**
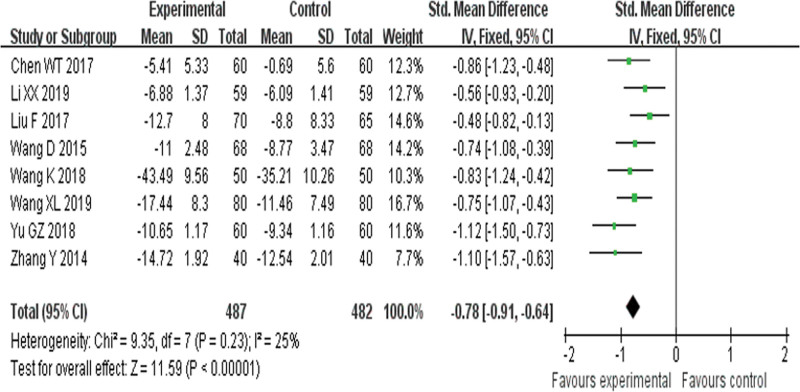
Forest plot of improvement of TCMSS. TCMSS = traditional Chinese medicine syndrome scores.

Eight RCTs^[19,23,25,26,32,33,35,37]^ reported TCMSS and found an obvious difference (*P* < .00001) between YQHX plus routine Western medicine treatment and routine Western medicine treatment alone on TCMSS (SMD = –0.78, 95% CI –0.91 to –0.64, 969 participants). The result indicated that YQHX combined with conventional Western drugs group was significantly better than conventional Western drugs group in the TCMSS and there was significant homogeneity among the 8 trials (*I*^2^ = 25%, *P* = .23) (Fig. 6).

##### 3.4.1.4. Minnesota Living with Heart Failure questionnaire.

**Figure 7.**
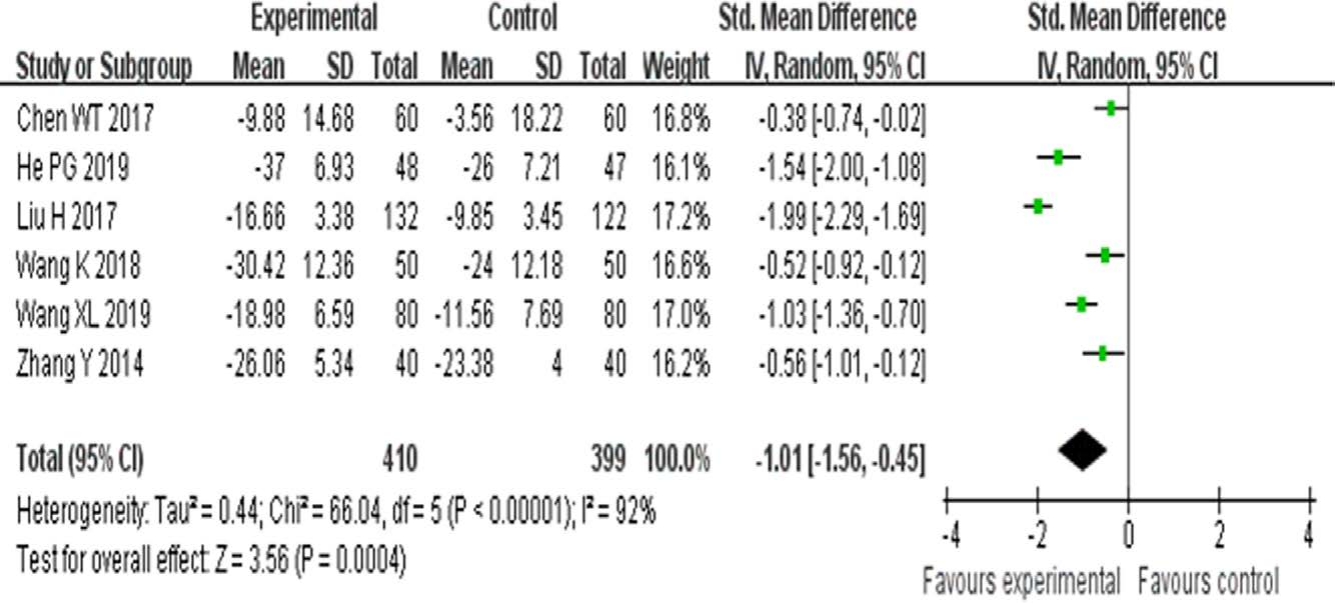
Forest plot of improvement of MLHFQ. MLHFQ = Minnesota Living with Heart Failure questionnaire.

Six RCTs^[23,25,27,30,33,37]^ reported MLHFQ and found an obvious difference (*P* = .0004) between YQHX plus routine Western medicine treatment and routine Western medicine treatment alone on MLHFQ (SMD = –1.01, 95% CI –1.56 to –0.45, 809 participants). The result indicated that YQHX combined with conventional Western drugs group was significantly better than conventional Western drugs group in the MLHFQ and there was significant homogeneity among the 6 trials (*I*^2^ = 92%, *P* < .00001) (Fig. 7).

##### 3.4.1.5. 6-Month readmission rate.

**Figure 8.**
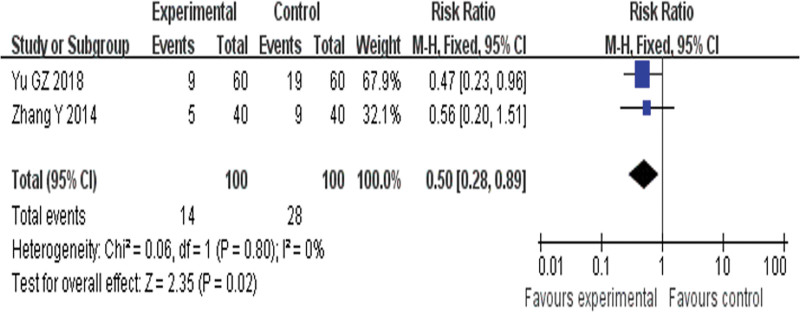
Forest plot of improvement of 6MRR. 6MRR = 6-month readmission rate.

Two RCTs^[26,37]^ reported 6MRR and found an obvious difference (*P* = .02) between YQHX plus routine Western medicine treatment and routine Western medicine treatment alone on 6MRR (RR = 0.50, 95% CI 0.28–0.89, 200 participants). The result indicated that YQHX combined with conventional Western drugs group was significantly better than conventional Western drugs group in the 6MRR and there was significant homogeneity among the 5 trials (*I*^2^ = 0%, *P* = .80) (Fig. 8).

##### 3.4.1.6. 6-Minute walk test.

**Figure 9.**
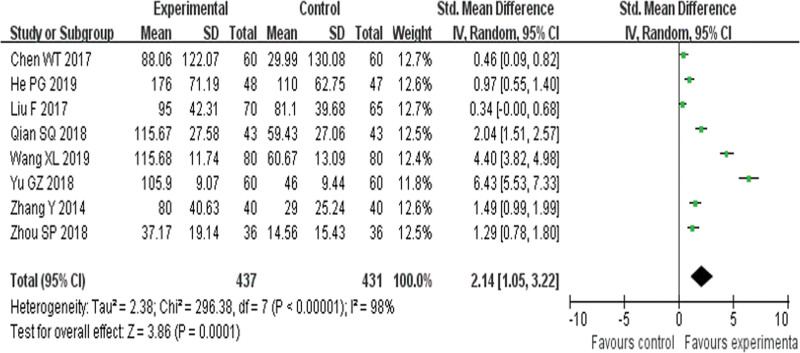
Forest plot of improvement of 6MWT. 6MWT = 6-minute walk test.

Eight RCTs^[19,22,23,26,27,31,33,37]^ reported 6MWT and found an obvious difference (*P* = .0001) between YQHX plus routine Western medicine treatment and routine Western medicine treatment alone on 6MWT (RR = 2.14, 95% CI 1.05–3.22, 868 participants). The result indicated that YQHX combined with conventional Western drugs group was significantly better than conventional Western drugs group in the 6MWT and there was significant homogeneity among the 8 trials (*I*^2^ = 98%, *P* < .00001) (Fig. 9).

#### 3.4.2. Serum biomarkers.

##### 3.4.2.1. BNP.

**Figure 10.**
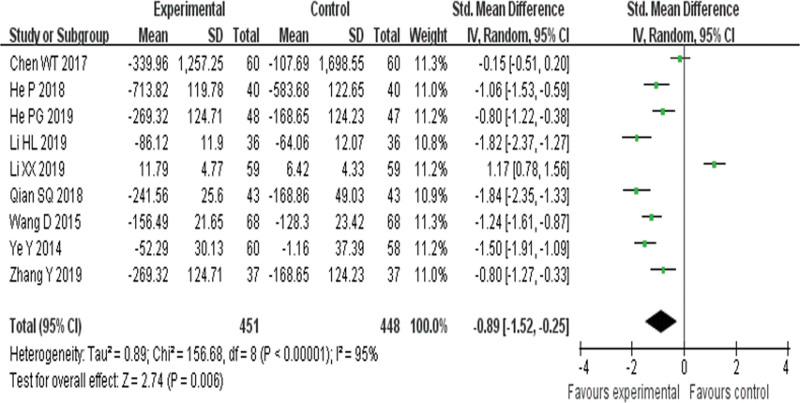
Forest plot of improvement of BNP. BNP = B-type natriuretic peptide.

Nine RCTs^[20,23,24,27,29,31,32,35,36]^ evaluated the effect of BNP and found BNP was significantly improved in the YQHX plus conventional drugs when compared with conventional drugs treatment alone (SMD = –0.89, 95% CI –1.52 to –0.25, 899 participants, *P* = .006). The result indicated that YQHX combined with conventional Western drugs group was significantly better than conventional Western drugs group in the improvement of BNP, and there was significant homogeneity among the 9 trials (*I*^2^ = 95%, *P* < .0001) (Fig. 10).

##### 3.4.2.2. NT-proBNP.

**Figure 11.**
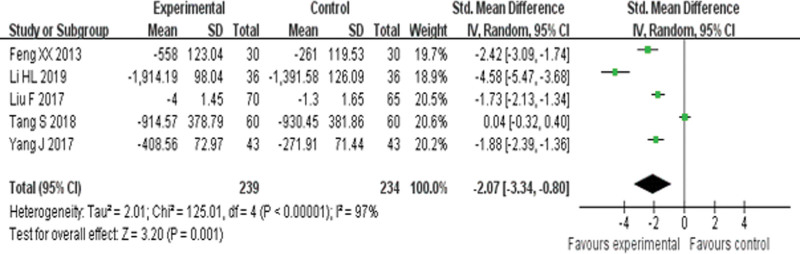
Forest plot of improvement of NT-proBNP. BNP = B-type natriuretic peptide.

Five RCTs^[19,21,28,29,34]^ reported NT-proBNP and found an obvious difference (*P* = .001) between YQHX plus routine Western medicine treatment and routine Western medicine treatment alone on NT-proBNP (SMD = –2.07, 95% CI –3.34 to –0.08, 473 participants). The result indicated that YQHX combined with conventional Western drugs group was significantly better than conventional Western drugs group in the NT-proBNP and there was significant homogeneity among the 5 trials (*I*^2^ = 97%, *P* < .00001) (Fig. 11).

##### 3.4.2.3. C-reactive protein.

**Figure 12.**
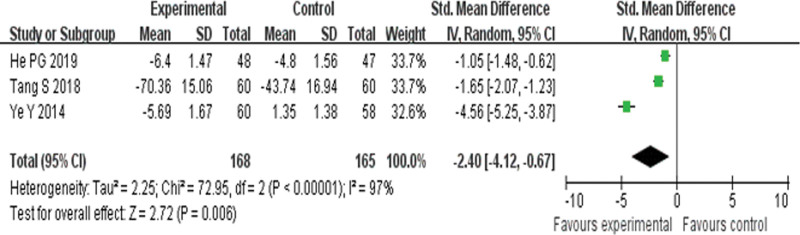
Forest plot of improvement of CRP. CRP = C-reactive protein.

Three RCTs^[20,21,27]^ reported CRP and found an obvious difference (*P* = .006) between YQHX plus routine Western medicine treatment and routine Western medicine treatment alone on CRP (SMD = –2.04, 95% CI –4.12 to –0.67, 333 participants). The result indicated that YQHX combined with conventional Western drugs group were significantly better than conventional Western drugs group in the CRP and there was significant homogeneity among the 3 trials (*I*^2^ = 97%, *P* < .00001) (Fig. 12).

#### 3.4.3. Cardiac function index.

##### 3.4.3.1. Left ventricular ejection fraction.

**Figure 13.**
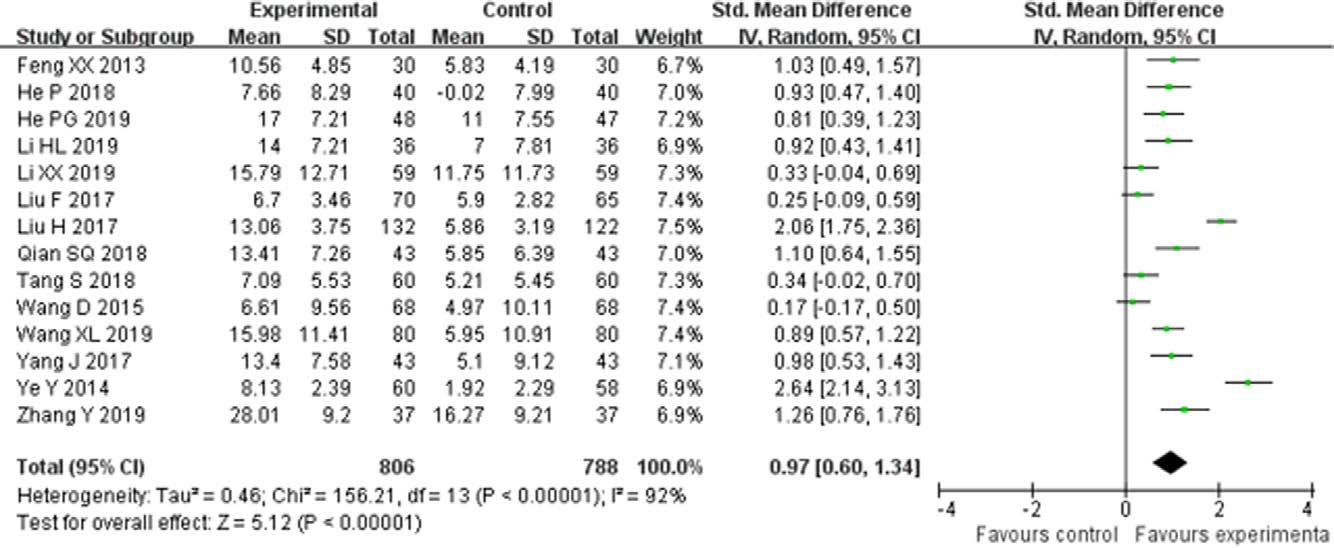
Forest plot of improvement of LVEF. LVEF = left ventricular ejection fraction.

Fourteen RCTs^[19–21,24,27–36]^ reported LVEF and found an obvious difference (*P* < .00001) between YQHX plus routine Western medicine treatment and routine Western medicine treatment alone on LVEF (RR = 0.97, 95% CI 0.60–1.34, 1594 participants). The result indicated that YQHX combined with conventional Western drugs group was significantly better than conventional Western drugs group in the LVEF and there was significant homogeneity among the 14 trials (*I*^2^ = 92%, *P* < .00001) (Fig. 13).

##### 3.4.3.2. Left ventricular end diastolic diameter.

**Figure 14.**
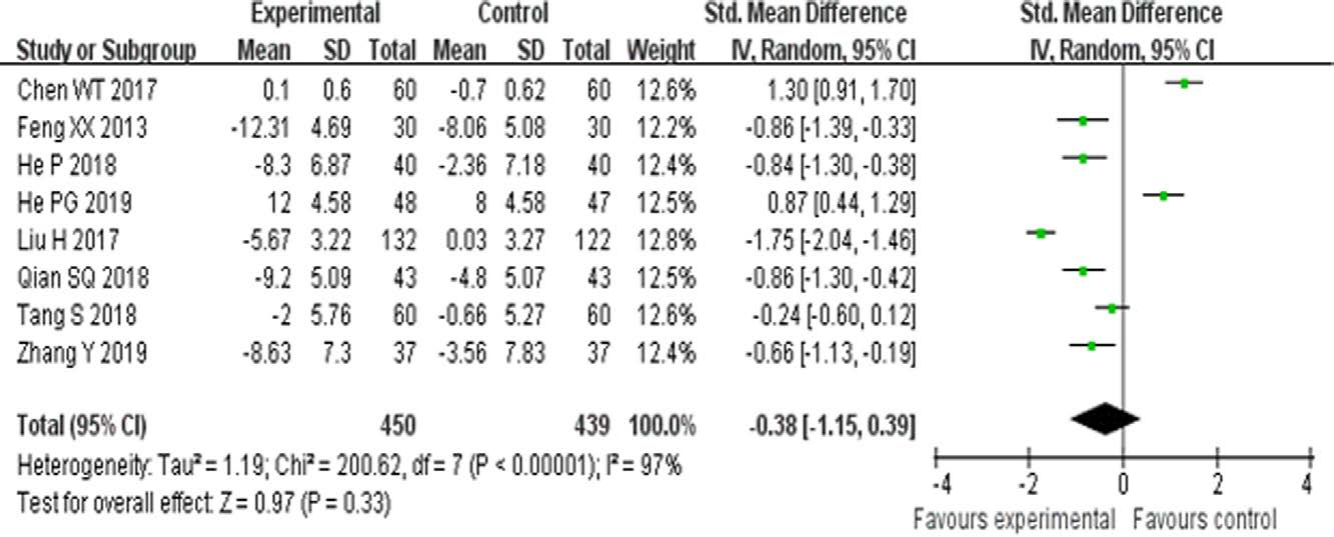
Forest plot of improvement of LVEDD. LVEDD = left ventricular end diastolic diameter.

Eight RCTs^[21,23,24,27,30,31,34,36]^ reported LVEDD and found no obvious difference (*P* = .33) between YQHX plus routine Western medicine treatment and routine Western medicine treatment alone on LVEDD (SMD = –0.38, 95% CI –1.15 to 0.39, 889 participants). The result indicated that YQHX combined with conventional Western drugs group was not significantly better than conventional Western drugs group in the LVEDD and there was significant homogeneity among the 8 trials (*I*^2^ = 0%, *P* < .00001) (Fig. 14).

##### 3.4.3.3. Stroke volume.

**Figure 15.**
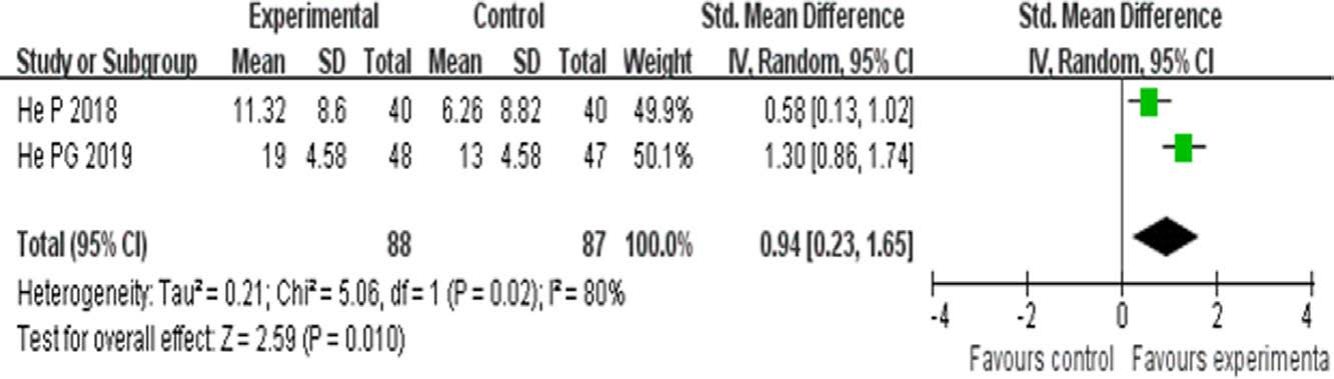
Forest plot of improvement of SV. SV = stroke volume.

Two RCTs^[24,27]^ reported SV and found an obvious difference (*P* = .02) between YQHX plus routine Western medicine treatment and routine Western medicine treatment alone on SV (SMD = 0.94, 95% CI 0.23–1.56, 175 participants). The result indicated that YQHX combined with conventional Western drugs group was significantly better than conventional Western drugs group in the SV and there was significant homogeneity among the 2 trials (*I*^2^ = 80%, *P* = .01) (Fig. 15).

##### 3.4.3.4. E/E′.

**Figure 16.**
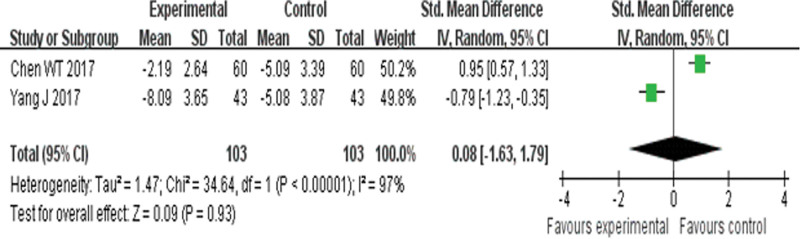
Forest plot of improvement of E/E′.

Two RCTs^[23,28]^ reported E/E′ and found no obvious difference (*P* = .93) between YQHX plus routine Western medicine treatment and routine Western medicine treatment alone on E/E′ (SMD = 0.80, 95% CI –1.63 to 1.97, 206 participants). The result indicated that YQHX combined with conventional Western drugs group was no significantly better than conventional Western drugs group in the E/E′ and there was significant homogeneity among the 2 trials (*I*^2^ = 97%, *P* < .00001) (Fig. 16).

#### 3.4.4. GRADE assessment.

Due to the poor methodology of the included studies and the obvious statistical heterogeneity among trials, quality of the evidence for all 5 outcomes (ORR, TCMSRR, BNP, NT-proBNP, LVEF) were “low” and “very low” according to the GRADE assessment (Table 3).

**Table 3:** Summary of finding table of Yiqihuoxue formula with conventional Western medicine for patients diagnosed with CHF.

Patient: Patients were diagnosed with CHF.						
Settings: Outpatient department/inpatient department.						
Intervention: Yiqihuoxue formula with conventional Western medicine.						
Control: Conventional Western medicine.						
Outcomes	Illustrative comparative risks* (95% CI)		Relative effect (95% CI)	No. of participants (studies)	Quality of the evidence (GRADE)	Comments
	Assumed risk	Corresponding risk				
	**Control**	**Yiqihuoxue**				
Overall response rate	**786 per 1000**	**952 per 1000**	**RR 1.21**	1410	⊕⊕⊝⊝	Further research is very likely to have an important impact on our confidence in the estimate of effect and is likely to change the estimate.
		(904–999)	(1.15–1.27)	(13 studies)	**Low** *	
TCM syndrome response rate	**727 per 1000**	**901 per 1000**	**RR 1.24**	367	⊕⊕⊝⊝	Further research is very likely to have an important impact on our confidence in the estimate of effect and is likely to change the estimate.
		(814–996)	(1.12–1.37)	(4 studies)	**low** *	
BNP		The mean BNP in the intervention groups was	**SMD –0.89**	899	⊕⊝⊝⊝	Further research is very likely to have an important impact on our confidence in the estimate of effect and is likely to change the estimate.
		**0.89 standard deviations lower**	(–1.52 to	(9 studies)	**Very low** *	
		(1.52–0.25 lower)	–0.25)			
NT-proBNP		The mean NT-proBNP in the intervention groups was	**SMD –2.07**	473	⊕⊝⊝⊝	Further research is very likely to have an important impact on our confidence in the estimate of effect and is likely to change the estimate.
		**2.07 standard deviations lower**	(–3.34 to –0.8)	(5 studies)	**Very low** *	
		(3.34–0.8 lower)				
LVEF		The mean LVEF in the intervention groups was	**SMD 0.97**	1594	⊕⊕⊝⊝	Further research is very likely to have an important impact on our confidence in the estimate of effect and is likely to change the estimate.
		**0.97 standard deviations higher**	(0.6–1.34)	(14 studies)	**Low** *	
		(0.6–1.34 higher)				

Bold values indicate the results of GRADE.

* There were serious limitations of methodological quality of included trials according to the risk of bias assessment.

BNP = B-type natriuretic peptide, CHF = chronic heart failure, CI = confidence interval, LVEF = left ventricular ejection fraction, RR = risk ratio, SMD = standardized mean difference, TCM = traditional Chinese medicine.

### 3.5. Publication bias

**Figure 17.**
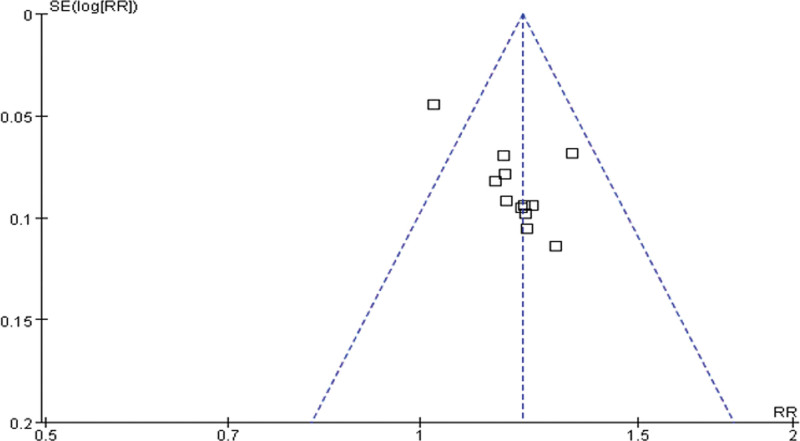
Funnel plot of the overall response rate.

In order to detect possible publication bias, we analyzed the 13 trials that compared YQHX plus routine Western medicine treatment with routine Western medicine treatment alone in terms of the ORRs with a fixed effects model. The funnel plot of ORR was asymmetrical, indicating the presence of publication bias (Fig. 17). The detection of publication bias was not available for other outcomes as the included trials were <10.

## 4. Discussion

### 4.1. YQHX and CWM treatment in CHF has clinical efficacy

CHF is a serious end-stage of various heart diseases which has high morbidity and mortality. It is a clinical syndrome characterized by insufficient blood perfusion, abnormal distribution of peripheral blood flow, and activation of neuroendocrine.^[2]^ Paroxysmal nocturnal dyspnea and edema are CHF’s typical clinical features. High jugular pressure, apical beat, and rale of lung are typical signs. It is difficult to recover and easy to recur.^[38]^ In terms of treatment, modern medicine has obvious advantages in the treatment of vasodilation, diuresis, and myocardial contractility; however, some patients with CHF have problems such as multiple organ damages, reduced tolerance of digitalis, hypotension, electrolyte disorder, and heavy economic burden due to the high cost of treatment.^[39]^ The treatment effect is often unsatisfactory, which provides a broad application space for the treatment of CHF by combining traditional Chinese and Western medicine. The basic pathogenesis of TCM syndrome differentiation is Qi deficiency and blood stasis; the method of invigorating qi and activating blood circulation (YQHX) is an important therapeutic principle of TCM in treating CHF. Results from previous studies showed that YQHX, as a complementary treatment, may improve the clinical symptoms, achieve the therapeutic effect, increase the exercise endurance, reduce the side effects of drugs, and increase the quality of life of CHF patients.^[9,11,40]^ Hence, the combination of YQHX prescription and CWM treatment in CHF has attracted more clinical attention, and the related research literature is gradually increasing, which provides a reliable basis for discussing the application value and guiding clinical practice of integrated traditional Chinese and Western medicine treatment in CHF.

### 4.2. Summary of evidence

YQHX, as a complementary treatment, may improve ventricular function and the quality of life of CHF patients. Nevertheless, the role of YQHX in the treatment of CHF is not fully clear. We aimed to provide the latest systematic review and meta-analysis to summarize the existing evidence of YQHX as an effective treatment for CHF. Unlike the previous meta-analysis, wherein the diagnosis was inappropriate due to outdated references hence the primary outcomes in the report were also inconsistent. With concerns on 19 trials, this meta-analysis about the ORR, TCMSRR showed that YQHX combined with CWM were more effective than CWM alone for CHF. Due to the poor methodological quality of the included trials and the insufficient number of trial participants, only limited evidence showed experimental group with YQHX may get better effect on natriuretic peptides (BNP or NT-proBNP), LEVF, TCMSS, 6MWT, MLHFQ, SV, 6MRR, and CRP, but the results had very significant heterogeneity. No significant differences were found between experimental groups and control groups on LVEDD and E/E′. At last, using of YQHX seemed safe and well tolerated for patients with CHF. In summary, although the strength of the evidence was low, we found potential effect of oral YQHX herbal preparations on improving some key symptoms in patients with CHF.

### 4.3. Possible explanations for the evidence

An important aim of treatment for CHF is to alleviate symptoms and improve well-being.^[41]^ According to this review, herbs or herbal prescriptions with the function of supplementing qi and activating blood circulation were commonly used to achieve this objective. Top 5 of most frequently used herbs were Huangqi (in 17 trials), Danshen (in 13 trials), Danggui (in 9 trials), Taoren (in 9 trials), and Chishao (in 8 trials). The modern pharmacological study confirmed that Huangqi could promote cell metabolism, improve the ability of myocardial hypoxia tolerance, reduce rennin-angiotensin and brain natriuretic peptide level, and improve heart function from many aspects.^[42]^ Danshen has the function of antioxidation and improving disorders of microcirculation.^[43]^ Danggui has the functions of antibacteria, antioxidation, and enhancing the immune function of the body.^[44]^ Taoren has abilities of anticoagulant, antithrombotic, and hemodynamics effects.^[45]^ Chishao has the functions of anticoagulant, antithrombotic, hypolipidemic, antiarteriosclerosis, and coronary artery dilation.^[46]^ The 5 herbs have pharmacological effects on treating heart failure in several fields.

### 4.4. Limitations of the review

Low levels of evidence in this review were mainly caused by the poor quality and small sample size of originally included trials. Only 5 out of 19 trials reported on how the participants were randomly assigned to the intervention groups. The other trials simply mentioned “randomization,” with none of the trials indicating the use of allocation concealment and blinding. Three of the trials specified follow-ups. This study suggested that YQHX can effectively improve the cardiac function of patients with CHF, but the results had very significant heterogeneity. The inconsistency of findings of herbal medicine’s effect on improving main outcomes of CHF among these trials further reduced the internal validity of the evidence. Although we searched both Chinese and English databases, all of the included trials were retrieved from Chinese literature, which may have introduced potential selection bias and limited the external generalization of the evidence.

## 5. Conclusions

Due to the insufficient quality of trials that were analyzed, this review could not authenticate the effectiveness of YQHX in treating CHF at the present time. Purposefully designed trials with high methodological quality are needed to validate the effect of YQHX for patients with CHF.

The results of the present systematic review will be disseminated to a variety of stakeholders interested in YQHX therapy to inform both the researchers for the further studies and clinical practice focused on CHF.

## Authors contributions

**Conceptualization:** Miao Zhang.

**Data curation:** Ming-Yue Sun.

**Formal analysis:** Ming-Yue Sun.

**Investigation:** Ma Min, Zheng-Zhi Wu.

**Methodology:** Miao Zhang.

**Project administration:** Feng-Qin Xu.

**Resources:** Ming-Yue Sun.

**Software:** Miao Zhang.

**Supervision:** Ma Min, Zheng-Zhi Wu.

**Validation:** Yu Jin.

**Visualization:** Yu Jin.

**Writing – original draft:** Miao Zhang.

**Writing – review & editing:** Feng-Qin Xu.
